# New Water-Soluble Carbamate Ester Derivatives of Resveratrol

**DOI:** 10.3390/molecules191015900

**Published:** 2014-10-01

**Authors:** Andrea Mattarei, Massimo Carraro, Michele Azzolini, Cristina Paradisi, Mario Zoratti, Lucia Biasutto

**Affiliations:** 1 Department of Chemical Sciences, University of Padova, Via F. Marzolo 1, 35131 Padova, Italy; E-Mails: andrea.mattarei@unipd.it (A.M.); cristina.paradisi@unipd.it (C.P.); 2 Department of Chemistry and Pharmacy, University of Sassari, Viale Vienna 2, 07100 Sassari, Italy; E-Mail: mcarraro@uniss.it (M.C.); 3 Department of Biomedical Sciences, University of Padova, Viale G. Colombo 3, 35121 Padova, Italy; E-Mails: michele.azzolini@gmail.com (M.A.); zoratti@mail.bio.unipd.it (M.Z.); 4 CNR Neuroscience Institute, Viale G. Colombo 3, 35121 Padova, Italy

**Keywords:** resveratrol, prodrugs, carbamate esters, solubility, poly(ethylene glycol), glucose

## Abstract

Low bioavailability severely hinders exploitation of the biomedical potential of resveratrol. Extensive phase-II metabolism and poor water solubility contribute to lowering the concentrations of resveratrol in the bloodstream after oral administration. Prodrugs may provide a solution—protection of the phenolic functions hinders conjugative metabolism and can be exploited to modulate the physicochemical properties of the compound. We report here the synthesis and characterization of carbamate ester derivatives of resveratrol bearing on each nitrogen atom a methyl group and either a methoxy-poly(ethylene glycol)-350 (mPEG-350) or a butyl-glucosyl promoiety conferring high water solubility. *Ex vivo* absorption studies revealed that the butyl-glucosyl conjugate, unlike the mPEG-350 one, is able to permeate the intestinal wall. *In vivo* pharmacokinetics confirmed absorption after oral administration and showed that no hydrolysis of the carbamate groups takes place. Thus, sugar groups can be attached to resveratrol to obtain soluble derivatives maintaining to some degree the ability to permeate biomembranes, perhaps by facilitated or active transport.

## 1. Introduction

Resveratrol has the potential to prevent, alleviate or slow the progression of a wide variety of illnesses, including cardiovascular disease, metabolic syndrome, cancer, ischemic injuries, cognitive decline, inflammatory ailments. It can enhance stress resistance and extend the lifespan of some model organisms. Studies dealing with the mechanisms underlying the bioactivity of resveratrol have been summarized in many reviews (e.g., [1,2,3,4,5,6,7,8]). Activation of the AMP-regulated kinase AMPK and of the NAD-dependent deacetylase Sirt-1, and the consequent effects on gene expression, are important features of the signaling network [9,10,11,12].

Bioavailability is fundamental for the full realization of the biomedical potential of nutraceuticals; many polyphenols have pharmacokinetic/pharmacodynamic and physicochemical properties that limit their chance of being developed into pharmaceutical products. The efficacy of orally administrated resveratrol depends on its absorption, metabolism, and tissue distribution. The distribution of resveratrol and its metabolites in organs has been investigated [13,14,15]. Only trace amounts (below 5 ng/mL, *i.e.*, 22 nM) of unchanged resveratrol could be detected in human plasma after a 25 mg oral dose [16]. A recent study of the distribution of pterostilbene (3,5-*O*-dimethylresveratrol) and pterostilbene sulfate in the rat has however shown that blood or plasma concentrations cannot be automatically considered a reliable index of total body levels [17]. The dose escalation approach has been investigated, covering a total dose range of 25–5,000 mg [18] but even at the highest doses the concentrations of resveratrol in plasma (peak: 550 ng/mL, *i.e.*, ~2.4 µM) seemed too low to provide a plausible explanation of effects. Indeed in enterocytes resveratrol is rapidly converted by conjugating enzymes to metabolites that are re-exported, largely to the intestinal lumen, by ABC transporters [19,20]. Liver sulfotransferases (SULTs) and glucuronosyltransferases (UGTs) then intervene on the molecules that have entered the circulation. Studies on *in vivo* bioavailability and metabolism of resveratrol indicate that glucuronides, sulfates and double-bond reduction products formed by gut microflora are major metabolites [13,18,21,22,23]. Marked differences in the relative relevance of these conjugative processes may be observed, depending on the species and also on factors such as gender or dosage (see, e.g., [24,25,26]). Most studies suggest sulfation to be the major modification in rodents, while glucuronidation appears more important in humans [17], but the significance of sulfation in humans may need reconsideration [14]. Metabolites themselves may have some degree of bioactivity (e.g., [27,28,29,30,31,32,33,34]) and the relative proportions of the two major sets of metabolites—sufates *vs.* glucuronides—may be a factor in determining overall bioactivity. While the contribution of these conjugates would be expected to depend on the specific cellular or molecular process being considered, in general their increased size, polarity and hydrophilicity may be seen as an obstacle for interaction with many enzymes.

Experimental data support the idea that sulfates and glucuronides of resveratrol may act as a sort of storage device, regenerating the aglycone through the action of sulfatases and glucuronidases [35,36,37]. Nonetheless, devising ways to increase levels and slow metabolic transformation of resveratrol after oral administration clearly is an important endeavor [38]. Many formulation-based approaches to overcome the solubility obstacle, improving stability and/or enhancing absorption and efficacy of resveratrol have been considered: inclusion into cyclodextrins [39,40], micro/nano emulsions [41,42,43], nano suspensions [44], micelles [45,46], nanostructured lipid carriers [47,48,49], liposomes [43,47,50], nanochannel delivery membrane systems [51], and various types of nanoparticulated carriers [48,52,53,54,55,56,57,58,59,60,61]. Another main strategy used to prevent drug metabolism and enhance bioavailability and effectiveness is based on the development of “prodrugs” [62,63]. In the case of resveratrol this would consist in protecting reactive sites (hydroxyl moieties) with removable groups (promoieties), thus opposing phase II metabolic processes. The prodrug approach is furthermore often used to introduce favorable physicochemical properties, in particular to modulate water solubility, via the promoieties. An ideal prodrug has both good absorption and bioconversion characteristics. The latter clearly depend on the type of chemical bond system used to link the promoiety to the “core” of the molecule.

**Scheme 1:**
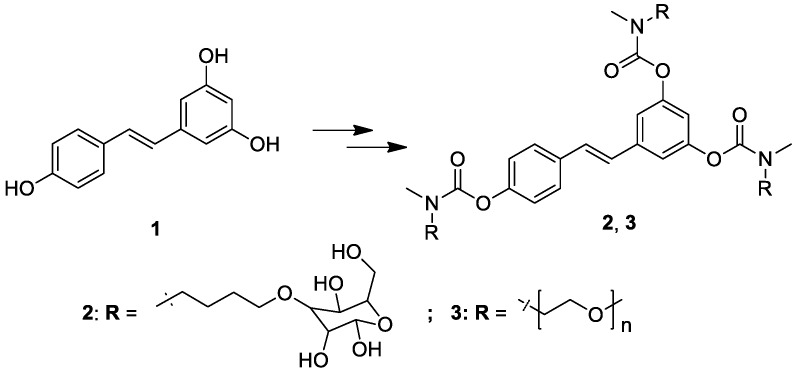
Chemical structures of resveratrol **1** and carbamoyl derivatives **2** and **3**.

Carbamate esters are one of the most popular types of prodrugs, used for example with duocarmycin [64], camptothecin [65], entacapone [66] and (−)-3-(3-hydroxyphenyl)-N-propylpiperidine [67]. We report here the synthesis and performance of *N,N*-disubstituted resveratrol carbamates. To improve solubility, butyl-glucosyl or methoxy-poly(ethylene glycol)-350 (mPEG-350; average molecular weight ~ 350 Da) groups were introduced as promoieties (Scheme 1).

Many prodrugs designed to increase water solubility involve the addition of an ionizable promoiety to the parent molecule. Because charged molecules have great difficulty crossing biological membranes, one must balance increased water solubility against a potentially decreased permeability. Based on this consideration we chose non-ionizable solubilizing groups. Conversion of a compound into a more hydrophilic one is not necessarily going to help absorption either, since the energetic barrier opposing diffusion through the lipid core of biological membranes is expected to become more important. However both groups we used, glucose and mPEG-350, have features that may provide increased solubility without unduly compromising absorption. Increased water solubility constitutes an obvious advantage also for administration routes other than oral, such as intravenous injection or slow-release implanted capsules.

It may be possible, by incorporating glucose into the promoiety, to take advantage of the presence of glucose transporters in the absorbing epithelium. While flavonoid or resveratrol intestinal uptake may also occur by simple diffusion through the enterocyte membrane, studies have implicated intestinal transporters of the SGLT and GLUT families in the uptake of glycosylated derivatives [68,69].

Attachment of poly(ethylene glycol) (PEG) moieties to therapeutic compounds (“pegylation”) may improve drug absorption and pharmacokinetics [70,71,72,73,74]. PEG is known to be non-toxic, non-antigenic and biocompatible and indeed the FDA has approved its use as a vehicle or base in foods, cosmetics and pharmaceuticals. In this work, mPEG-350 was chosen as a promoiety to modify resveratrol because it can increase both water and lipid solubility of a drug and we reasoned that the latter effect may facilitate the penetration of resveratrol through biological membranes.

## 2. Results and Discussion

### 2.1. Synthesis

**Scheme 2:**
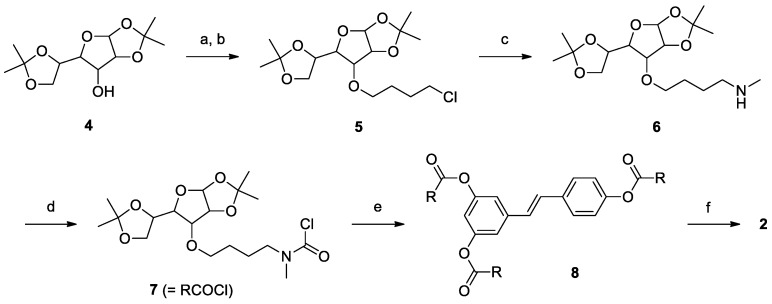
Synthesis of compound **2**.

Derivatives **2–3** (Scheme 1) were obtained through condensation of resveratrol (**1**) with the appropriate carbamoyl chloride. The synthesis of **2** is outlined in Scheme 2. The starting material for the glucosyl carbamate was commercially available 1,2:5,6-di-*O*-isopropylidene-α-d-glucofuranose (**4**) which was allowed to react with an excess of 1-bromo-4-chlorobutane in the presence of sodium hydride to give **5** in fair yield through Williamson etherification. In the second step, compound **5** was treated with methylamine under microwave irradiation to obtain the secondary amine **6** in a short reaction time. This intermediate was allowed to react with triphosgene under mild conditions to give the carbamoyl chloride (**7**) which was used without further purification in the subsequent step. Compound **7** was added in slight excess to resveratrol (**1**) in pyridine and allowed to react at reflux to obtain the carbamate derivative **8** in good yield. The last step consisted in the removal of the acetonide protecting groups from the glucofuranosyl rings, freeing the hydroxyl functions necessary to enhance the solubility in water of the final product (**2**). The reaction was performed in trifluoroacetic acid:water, 9:1, at room temperature. Under these conditions the carbamate group is stable and the desired product was obtained in excellent yield.

*Reagents and conditions:* (a) NaH, DMF, r.t., 30 min; (b) Br(CH_2_)_4_Cl, 40 °C, 24 h; (c) CH_3_NH_2_, EtOH, Microwave 150 °C, 30 min; (d) triphosgene, pyridine, DCM, 0 °C to r.t., 45 min; (e) DMAP, **1**, pyridine, reflux, overnight; (f) trifluoroacetic acid : water 9:1, r.t., 1 h.

**Scheme 3:**
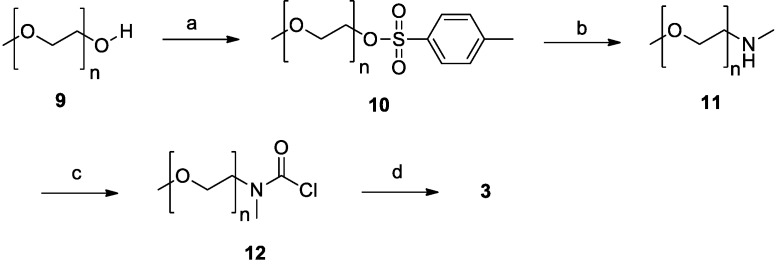
Synthesis of compound **3**.

The synthesis of derivative **3** (Scheme 3) was more straightforward, since there was no need to introduce a spacer or for a deprotection step. The starting material was a simple and cheap methoxy-poly(ethylene glycol) with an average molecular weight of 350 Da (**9**), possessing one free hydroxyl group which was esterified with tosyl chloride to obtain compound **10**. The tosylate group of **10** was easily displaced in the second step by methylamine under microwave irradiation giving the secondary amine **11** in nearly quantitative yield. Finally, amine **11** was treated with triphosgene to give the carbamoyl chloride **12**, which in turn was allowed to react with resveratrol to afford the desired product **3**.

*Reagents and conditions:* (a) 1. NaOH, H_2_O/THF, 0 °C, 2. TosCl, 5 °C, 2 h; (b) CH_3_NH_2_, EtOH, Microwave 150 °C, 30 min; (c) triphosgene, pyridine, DCM, 0 °C to r.t., 45 min; (d) DMAP, **1**, pyridine, reflux, overnight.

### 2.2. Solubility

Both derivatives displayed good water solubility (>50 mM). This represents a strong increase with respect to resveratrol, the reported solubility for which ranges from ≤1 µM to 300 µM [75,76,77,78]. As can be noticed, these data from the literature are very variable, probably because of the difficulty in determining accurate solubility data for compounds that can form colloids.

### 2.3. Permeation across Rat Intestine

We assayed transport of the derivatives across explanted rat jejunum segments using Ussing-type chambers. This system allows the study of transepithelial transport separately from other processes taking place in the intestinal lumen, in blood, or in the liver. In these experiments, the same amount of resveratrol or derivative was loaded on the apical side; both apical and basolateral chambers were then analyzed after 2.5 h (see Experimental Sections for details).

In agreement with our previous observations [78,79], resveratrol reached the basolateral compartment mainly (≥90%) in the form of phase II metabolites (sulfate(s) and glucuronide(s)); basolateral species collectively accounted for 0.6%–1.5% of the amount placed initially in the apical side chamber. In the same experimental setup and conditions, a similar translocation (1.7 ± 0.7%) was observed for derivative **2**; in this case, however, only the intact derivative was detected in the basolateral chamber; resveratrol and its metabolites were below the detection threshold, and no products deriving from partial deprotection of the hydroxyl groups (hydrolysis of one or two of the three carbamate groups) could be detected either. Somewhat to our surprise, no compounds with a stilbenoid structure could be detected in the basolateral chamber when derivative **3** was placed in the apical one. No further experiments were conducted with this compound.

Passage of the glucosyl-derivative **2** across the intestinal wall is remarkable, since it is too hydrophilic to undergo passive diffusion through cell membranes. A possible explanation may be the involvement of a facilitated or active transport mechanism, possibly mediated by glucose transporters. Dietary glucose crosses the apical membrane of the enterocyte by the Na^+^/glucose cotransporter (SGLT1) and exits across the basolateral membrane through the facilitative transporter GLUT2. Before a meal, the concentration of glucose in the lumen is very low. Any glucose is rapidly captured by SGLT1, which is ideal for this purpose, being a low-capacity, high-affinity transporter and the only transporter capable of moving glucose against a concentration gradient. GLUT2 is a high-capacity, low-affinity facilitative transporter that equilibrates glucose between plasma and enterocyte. We thus investigated the absorption of derivative **2** through excised rat jejunum in the presence of different concentrations and combinations of inhibitors of SGLT-1 (phlorizin) and GLUT-2 (phloretin and cythochalasin B). To minimize the possibility of competitive inhibition by medium glucose, we also decreased glucose concentration in the assay medium from ~55 to 1 mM, substituting it with mannitol, and/or increased the concentration of derivative **2** to levels close to that of glucose (0.5 *vs* 1 mM).

After a meal, there is a high effective glucose concentration at the surface of the apical membrane. The initial glucose transport across the apical membrane results in rapid insertion of GLUT2 into the apical membrane from intracellular vesicles underlying the membrane. Apical GLUT2 is now the major pathway of absorption. Then, when the glucose concentration in the lumen falls, the whole signaling system is reversed so that GLUT2 is inactivated and traffics away from the apical membrane to restore the situation before a meal [80,81]. To determine if the administration of glucose could enhance the absorption of derivative **2**, thus providing evidence for a role of GLUT2 in its transport, we performed transport experiments using the intestine from non-fasted rats, and from rats who had received glucose in their drinking water (20 g/L) in the 24 h prior to the experiment.

The extents of translocation under the various conditions mentioned above turned out not to differ significantly one from the others. Dynamic trafficking of GLUT-2 [82] may contribute to the observed variability: its levels in *ex vivo* intestinal preparations can be greatly reduced compared to the *in vivo* situation, because GLUT-2 traffic to the brush border of enterocytes is regulated by endogenous hormones through PKC βII activation, and this process is inactivated upon intestine explantation. A variable contribution by paracellular absorption may also take place; this can be altered/increased by stretching of the intestinal segment during the assembly of Ussing chambers, which in turn depends on the particular anatomical features of each explanted intestine. To gain more information on the absorption of derivative **2**, we thus performed *in vivo* pharmacokinetic experiments.

### 2.4. In Vivo Pharmacokinetics

**Figure 1:**
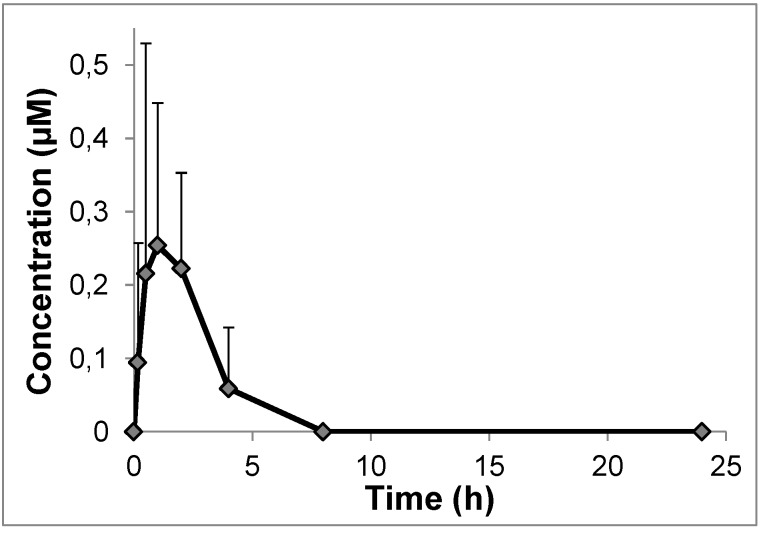
Results of pharmacokinetic experiments in rats with derivative **2**. Blood concentration *vs* time profile. Mean values ± st. dev. (*n* = 5). To compare these results with the pharmacokinetic profile obtained administering resveratrol itself, please see [78].

Oral administration by gavage of derivative **2** to rats resulted in low concentrations of the derivative in the bloodstream during the 4 hours following administration; maximum levels were reached after about 60 min (Figure 1). This suggests that the paracellular pathway may not be involved in uptake, but the mechanism of absorption remains to be defined. Besides sugar carriers, other candidates are provided by the families of Organic Anion Transporter Polypeptides (OATPs) (e.g., [83,84,85,86]) and/or Organic Cation Transporters (OCTs) [87,88].

Neither resveratrol nor its common metabolites were present in detectable amounts either in the basolateral chamber in *ex vivo* assays or in the blood of rats after oral administration. It follows that while **2** can be transported across the intestinal wall, although with a low efficiency, it resists the action of hydrolytic enzymes in the gut, gut wall, blood and liver. Also derivative **3** exhibited complete stability at physiological pH values and in blood; one can thus conclude that *N,N*-disubstituted carbamate derivatives are stable under the conditions of interest here. The ideal bond in prodrugs needs on the other hand to have a finite lifetime *in vivo*. The stability of the carbamate ester group can be fine-tuned by acting on the substitution pattern of the N atom: *N*-monosubstituted carbamates are known to be less stable than *N,N*-disubstituted ones [89].

## 3. Experimental Section

### 3.1. Materials, Reagents and Standard Procedures

Resveratrol was purchased from Waseta Int. Trading Co. (Shangai, China). Other starting materials and reagents were purchased from Sigma-Aldrich, Fluka, Merck-Novabiochem, Riedel de Haen, J.T. Baker, Cambridge Isotope Laboratories Inc., Acros Organics, Carlo Erba and Prolabo, and were used as received. ^1^H-NMR spectra were recorded with a Bruker AC250F spectrometer operating at 250 MHz. Chemical shifts (δ) are given in ppm relative to the signal of the solvent. HPLC/ESI-MS analyses and mass spectra determinations were performed with a 1100 Series Agilent Technologies system, equipped with a binary pump (G1312A) and a MSD SL Trap mass spectrometer (G2445D SL) with an ESI source. Reaction intermediates and final purified products were injected as solutions in acetonitrile; elution was carried out with a water:acetonitrile 1:1 mixture containing 0.1% formic acid. TLCs were run on silica gel supported on plastic (Macherey-Nagel Polygram^®^SIL G/UV_254_, silica thickness 0.2 mm), or on silica gel supported on glass (Fluka) (silica thickness 0.25 mm, granulometry 60 Å, medium porosity) and visualized by UV detection or KMnO_4_ oxidation. Flash chromatography was performed on silica gel (Macherey-Nagel 60, 230–400 mesh granulometry (0.063–0.040 mm)) under air pressure. Solvents were analytical or synthetic grade and were used without further purification. Elemental analyses were carried out using a Fisons-EA-1108 CHNS-O Element Analyzer (Thermo Scientific).

### 3.2. Synthesis

*1,2,5,6-Di-O-isopropylidene-3-(4-chlorobutoxy)-α-d-glucofuranose* (**5**): NaH (0.31 g, 7.7 mmol, 2.0 eq., 60% in mineral oil) was washed three times with 5 mL of *n*-hexane. The suspension was decanted after each wash and *n*-hexane traces were removed under reduced pressure. Anhydrous DMF (15 mL) was then added and the suspension was stirred for 5 min. A solution of 1,2,5,6-Di-*O*-isopropylidene-α-d-glucofuranose (**4**, 1.00 g, 3.8 mmol, 1.0 eq.) in anhydrous DMF (15 mL) was then added dropwise. After stirring for 30 min under nitrogen flow, a solution of 1-bromo-4-chlorobutane (2.63 g, 15.4 mmol, 4.0 eq.) in anhydrous DMF (5 mL) was added dropwise and the mixture was vigorously stirred for 24 hours at 40 °C. The reaction mixture was then diluted in CH_2_Cl_2_ (150 mL) and washed with 0.3 N HCl (5 × 100 mL). The organic layer was dried over MgSO_4_ and filtered. The solvent was evaporated under reduced pressure and the residue was purified by column chromatography (CH_2_Cl_2_:EtOAc 9:1) to give **5** (0.81 g, 60% yield) as a pale yellow oil. ^1^H-NMR (CDCl_3_) δ (ppm) = 5.87 (d, *J* = 3.7 Hz, 1H, -CH-), 4.53 (d, *J* = 3.8 Hz, 1H, -CH-), 4.32–4.24 (m, 1H, -CH-), 4.12–4.06 (m, 2H, -CH_2_-), 4.00–3.95 (m, 1H, -CH-), 3.86–3.44 (m, 5H, -CH-, -O-CH_2_-, -CH_2_-Cl), 1.84–1.60 (m, 4H, -CH_2_-CH_2_-), 1.49, 1.41, 1.34, 1.31 (4 s, 12H, 4 × -CH_3_); ^13^C-NMR (CDCl_3_) δ (ppm) = 111.76, 108.98, 105.25, 83.05, 82.12, 81.21, 72.34, 69.54, 67.39, 44.76, 29.26, 26.99, 26.80, 26.77, 26.21, 25.34; ESI-MS (ion trap): *m/z* 351 [M+H]^+^. Anal. calcd. for C_16_H_27_ClO_6_: C, 54.78; H, 7.76 Found: C, 54.69; H, 7.71.

*1,2,5,6-Di-O-isopropylidene-3-(4-N-methyl-N-butoxyamino)-α-d-glucofuranose* (**6**): In a sealed microwave reactor **5** (1.99 g, 5.7 mmol, 1.0 eq.) was added to methylamine solution (33 wt. % in absolute EtOH, 25 mL, 198.5 mmol, 35.0 eq.). The reaction mixture was stirred for 30 min under microwave irradiation (150 °C, 150 W). The solvent was evaporated under reduced pressure and the residue was purified by column chromatography (CH_2_Cl_2_/MeOH 8.5:1.5) to give **6** (1.78 g, 91% yield) as a white solid. ^1^H-NMR (DMSO-*d*_6_) δ (ppm) = 5.83 (d, *J* = 3.7 Hz, 1H, -CH-), 4.63 (d, *J* = 3.8 Hz, 1H, -CH-), 4.27–4.20 (m, 1H, -CH-), 4.04–3.94 (m, 2H, -CH_2_-), 3.84–3.74 (m, 2H, -CH_2_-), 3.64–3.56 (m, 1H, -CH-), 3.46–3.32 (m, 4H, -CH-, -NH-CH_3_), 2.90–2.76 (m, 2H, -NH-CH_2_-), 2.43 (br, 1H, -NH-), 1.72–1.48 (m, 4 H, -CH_2_-CH_2_-), 1.39, 1.32, 1.27, 1.25 (4 s, 12H, 4 × -CH_3_); ^13^C-NMR (DMSO-*d*_6_) δ (ppm) = 110.78, 107.98, 104.67, 81.56, 81.48, 80.28, 72.29, 68.72, 65.93, 47.83, 32.18, 26.65, 26.61, 26.22, 26.08, 25.32, 22.26; ESI-MS (ion trap): *m/z* 346 [M+H]^+^. Anal. calcd. for C_17_H_31_NO_6_: C, 59.11; H, 9.05; N, 4.05 Found: C, 59.15; H, 9.01; N, 4.11.

*3,4',5-Tri-[1,2,5,6-Di-O-isopropylidene-α-d-glucofuranose-3-(4-N-methyl-N-butoxy)]-resveratrol carbamate* (**8**): A solution of **6** (2.03 g, 5.9 mmol, 1.0 eq.) and pyridine (5.0 mL) in dry CH_2_Cl_2_ (20 mL) was slowly added to triphosgene (1.74 g, 5.9 mmol, 1.0 eq.) in dry CH_2_Cl_2_ (20 mL) at 0 °C. The mixture was stirred at room temperature for 45 min followed by addition of 0.1 N HC1 (50.0 mL). The organic layer was separated, and washed 4 × 100 mL with 0.1 N HCl and then dried over MgS0_4_, filtered and concentrated under reduced pressure. The residue was then filtered through a short silica-gel column (CH_2_Cl_2_:EtOAc 8.25:1.75) and the eluate concentrated to give **7** as a pale yellow oil, which was used immediately without further purification. Compound **7** (1.70 g, 4.2 mmol, 5 eq.) was then added to a solution of resveratrol (**1**, 0.190 g, 0.8 mmol, 1 eq.) and DMAP (0.407 g, 3.3 mmol, 4 eq.) in pyridine (5 mL) and the resulting solution was vigorously stirred for 16 hours at 135 °C. The reaction mixture was then diluted in EtOAc (150 mL) and washed with 0.5 N HCl (5 × 100 mL). The organic layer was dried over MgSO_4_ and filtered. The solvent was evaporated under reduced pressure and the residue was purified by column chromatography (CH_2_Cl_2_:EtOAc 5.5:4.5) to give **8** (0.86 g, 76% yield) as a white solid. ^1^H-NMR (CDCl_3_) δ (ppm) = 7.46 (d, *J* = 8.6 Hz, 2H, 2 × Ar-H), 7.17–6.92 (m, 6H, 6 × Ar-H), 6.89–6.81 (m, 1H, Ar-H), 5.87 (d, *J* = 3.7 Hz, 3H, 3 × -CH-), 4.58–4.50 (m, 3H, 3 × -CH-), 4.35–4.27 (m, 3H, 3 × -CH-), 4.15–3.96 (m, 9H, 3 × -CH-, 3 × -CH_2_-), 3.89–3.84 (m, 3H, 3 × -CH-), 3.72–3.52 (m, 6H, 3 × -CON(CH_3_)-CH_2_-), 3.48–3.32 (m, 6H, 3 × -O-CH_2_-), 3.10–2.96 (2 s, 9H, 3 × -CON(CH_3_)-, 1.77–1.55 (m, 12H, 3 × -CH_2_-CH_2_-), 1.49, 1.42, 1.35, 1.31 (4 s, 36H, 12 × -CH_3_); ^13^C-NMR (CDCl_3_) δ (ppm) = 154.73, 154.42, 151.07, 150.78 127.61, 122.09, 116.59, 111.89, 111.81, 109.94, 109.10, 105.38, 82.57, 82.28, 81.28, 72.58, 70.18, 70.15, 67.43, 49.19, 49.09, 31.09, 26.96, 26.34, 25.57; ESI-MS (ion trap): *m/z* 1365 [M+H]^+^. Anal. calcd. for C_68_H_99_N_3_O_24_: C, 60.84; H, 7.43; N, 3.13 Found: C, 60.77; H, 7.40; N, 3.08.

*3,4',5-Tri-[α/β-d-glucopyranose-3-(4-N-methyl-N-butoxy)]-resveratrol carbamate* (**2**): A solution of **8** (0.30 g, 0.2 mmol), trifluoroacetic acid (1.8 mL), and water (0.2 mL) was stirred for 1.5 hours at room temperature. The reaction mixture was then added dropwise to diethyl ether (10 mL) under stirring and the precipitate was centrifuged (1000 g, 5 min). The solvent was decanted and the precipitate was washed three times with diethyl ether (10 mL) in order to eliminate residual traces of trifluoroacetic acid. The resulting solid was dissolved in water (5 mL) and lyophilized to give **2** (0.23 g, 93% yield) as a bright white solid. ^1^H-NMR (DMSO-*d*_6_) δ (ppm) = 7.61 (d, *J* = 8.6 Hz, 2H, 2 × Ar-H), 7.37–6.11 (m, 6H, 6 × Ar-H), 6.89–6.81 (m, 1H, Ar-H), 4.89 (d, *J* = 3.7 Hz, 3H, 3 × -CH-), 4.28 (d, *J* = 7.0 Hz, 3H, 3 × -CH-), 3.79–2.86 (m, 36H), 1.75–1.43 (m, 12H, 3 × -CH_2_-CH_2_-); ^13^C-NMR (CDCl_3_) δ (ppm) = 153.69, 153.48, 151.82, 150.94, 138.76, 133.57, 132.62, 127.39, 126.63, 122.16, 96.84, 92.27, 85.16, 81.90, 76.64, 74.51, 72.03, 71.44, 71.43, 71.41, 71.40, 71.38, 69.85, 69.83, 69.66, 69.65, 61.00, 48.54, 34.43, 34.13, 26.99, 26.97; ESI-MS (ion trap): *m/z* 1124.5 [M+H]^+^. Anal. calcd. for C_50_H_75_N_3_O_24_: C, 54.49; H, 6.86; N, 3.81 Found: C, 54.54; H, 6.84; N, 3.71.

*(Methoxypolyethylene glycol 350)-p-toluenesulfonate* (**10**): A solution of sodium hydroxide (2.29 g, 57.3 mmol, 2.0 eq.) in water (20 ml) and mPEG-350 (**9**, 10.0 g, 28.6 mmol, 1 eq.) in THF (20 mL) was cooled in an ice–water bath with stirring and tosyl chloride (10.9 g, 57.3 mmol, 2 eq.) in THF (20 ml) was added dropwise over 2 hours. The reaction mixture was stirred for an additional 2 hours at 5 °C, poured into ice–water (100 mL) and extracted with CH_2_Cl_2_ (3 × 100 mL). The combined organic extracts were dried over MgSO_4_ and filtered. The solvent was evaporated under reduced pressure and the residue was purified by column chromatography (CHCl_3_:MeOH 9.9:0.1 and increasing methanol content) to give **10** (11.03 g, 77% yield) as a colourless oil. ^1^H-NMR (CDCl_3_) δ (ppm) = 7.78 (d, *J* = 8.3 Hz, 2H, 2 × Ar-H), 7.33 (d, *J* = 8.3 Hz, 2H, 2 × Ar-H), 4.15 (t, *J* = 4.90 Hz, 2H, -(SO_2_)O-CH_2_-), 3.70–3.51 (m, 30H, 7 × -O-CH_2_-CH_2_-O- + -O-CH_2_-), 3.37 (s, 3H, -O-CH_3_), 2.44 (s, 3H, Ar-CH_3_); ^13^C-NMR (CDCl_3_) δ (ppm) = 144.74, 132.90, 129.77, 127.93, 71.87, 70.68, 70.54, 70.50, 70.46, 69.20, 68.62, 58.98, 21.62; ESI-MS (ion trap): *m/z* 495 [M_(*n* = 7)_+H]^+^.

*N-methyl-N-(methoxypolyethylene glycol 350) amine* (**11**): In a sealed microwave reactor **10** (5.43 g, 10.1 mmol, 1.0 eq.) was added to methylamine solution (33 wt. % in absolute ethanol, 44 mL, 353.5 mmol, 35.0 eq.). The reaction mixture was stirred for 30 min under microwave irradiation (150 °C, 150 W). After evaporation of the solvent, the residue was dissolved in 5% HCl (20 mL) and extracted with CHCl_3_ (3 × 20 mL). The CHCl_3_ extracts were washed separately with 5% HCl (2 × 10 mL) and all the HCl fractions were combined, 30% sodium hydroxide (15 mL) was added and the resulting solution was extracted with chloroform (3 × 25 mL). The chloroform extracts were then washed separately with water (10 mL). The organic layer was dried over MgSO_4_ and filtered. The solvent was then evaporated under reduced pressure to give **11** as a pale yellow oil (3.60 g, 98% yield). ^1^H-NMR (CDCl_3_) δ (ppm) = 3.70–3.51 (m, 30H, 7 × -O-CH_2_-CH_2_-O- + -O-CH_2_-), 3.36 (s, 3H, -O-CH_3_), 2.74 (t, *J* = 4.90 Hz, 2H, -NH-CH_2_-), 2.43 (s, 3H, -NH-CH_3_), 2.16 (s, 1H, -NH-); ^13^C-NMR (CDCl_3_) δ (ppm) = 71.86, 70.53, 70.50, 70.42, 70.24, 70.07, 58.97, 51.11, 36.15; ESI-MS (ion trap): *m/z* 354 [M_(*n* = 7)_+H]^+^.

*3,4',5-Tri-[N-methyl-N-(methoxypolyethylene glycol 350)]-resveratrol carbamate* (**3**): A solution of **11** (3.70 g, 10.2 mmol, 1.0 eq.) and pyridine (5.0 mL) in dry CH_2_Cl_2_ (20 mL) was slowly added to triphosgene (3.02 g, 10.2 mmol, 1.0 eq.) in dry CH_2_Cl_2_ (34 mL) at 0 °C. The mixture was stirred at room temperature for 45 min, followed by addition of 0.1 N HCl (50 mL). The organic layer was separated, washed with 0.1 N HCl (4 × 100 mL) and then dried over MgS0_4_, filtered and concentrated under reduced pressure. The residue was then filtered through a short silica-gel column (CH_2_Cl_2_:MeOH 9:1) and the eluate concentrated to give **12** as a pale yellow oil, which was used immediately without further purification. **12** (1.77 g, 4.2 mmol, 5 eq.) was added to a solution of resveratrol (**1**, 0.190 g, 0.8 mmol, 1 eq.) and DMAP (0.407 g, 3.3 mmol, 4 eq.) in pyridine (5 mL) and the resulting solution was vigorously stirred for 16 hours at 135 °C. The reaction mixture was then diluted in EtOAc (150 mL) and washed with 0.5 N HCl (5 × 100 mL). The organic layer was dried over MgSO_4_ and filtered. The solvent was evaporated under reduced pressure and the residue was purified by column chromatography (eluent: ACN) to give **3** (0.88 g, 76% yield) as a colourless oil. ^1^H-NMR (CDCl_3_) δ (ppm) = 7.45 (d, *J* = 8.6 Hz, 2H, 2 × Ar-H), 7.26-6.97 (m, 6H, 6 × Ar-H), 6.92–6.83 (m, 1H, Ar-H), 3.71-3.52 (m, 9H, 21 × -O-CH_2_-CH_2_-O-, 3 × -O-CH_2_-, 3 × -CON(CH_3_)-CH_2_-), 3.37 (s, 9H, 3 × -O-CH_3_), 3.15–3.07 (2 s, 9H, 3 × -NH-CH_3_), 2.16 (s, 1 H, -NH-); ^13^C-NMR (CDCl_3_) δ (ppm) = 154.33, 154.17, 151.93, 151.07, 139.11, 133.99, 129.29, 129.23, 127.39, 127.16, 127.13, 121.94, 116.43, 116.41, 71.88, 70.53, 70.48, 69.71, 69.32, 59.03, 59.00, 49.12, 49.07, 48.98, 36.04; ESI-MS (ion trap): *m/z* 1367 [M_(*n* = 7)_+H]^+^.

### 3.3. Permeation Studies with Rat Intestinal Segments (Ex Vivo)

All experiments involving animals were performed with the permission and supervision of the University of Padova Ethical Committee for Experimentation on Animals (CEASA) and Central Veterinary Service, in compliance with Italian Law DL 116/92, embodying UE Directive 86/609.

Intestine was excised from 18 h fasted rats and transferred into a saline solution (154 mM NaCl in water) at 37 °C. The jejunum was cut into 1 cm long strips, opened longitudinally, rinsed free of luminal content and mounted in Ussing-type chambers. Apical and basolateral compartments were filled with 1 mL each of oxygenated HEPES buffer (248 mM NaCl, 55.3 mM glucose, 50 mM NaHCO_3_, 9.9 mM KCl, 1.9 mM MgSO_4_, 40 mM HEPES, pH 6.8), and incubated in a water bath at 37 °C until all chambers were assembled (approximately 20 min). The buffer was then removed and substituted with 1 mL of a 20 µM solution (in the same buffer) of the compound to be tested on the apical side (dilution from a 20 mM stock solution), and with 1 mL of fresh HEPES buffer on the basolateral one. Stock solutions of the compounds were prepared in water (for the derivatives) or in DMSO (for resveratrol, due to its limited water solubility). During the experiment, oxygen was continuously bubbled in each basolateral compartment. An aliquot of the initial apical solutions was incubated separately at 37 °C for the period of the experiment, to verify the stability of each compound in the absence of jejunum. At the end of the experiment (2.5 h), 800 µL of chamber contents from apical and basolateral sides were collected and mixed with 8 µL of 100 mM ascorbic acid in water and 8 µL of 6 M acetic acid. The samples were then centrifuged (12,000× *g*, 7 min, 4 °C), and supernatants were frozen and maintained at −20 °C until HPLC-UV analysis (see below).

### 3.4. Pharmacokinetic Studies

Derivative **2** was administered to overnight-fasted male Wistar rats from the facility of the Department of Biomedical Sciences as a single intragastric dose (88 µmol/kg, dissolved in 250 µL water). Blood samples were obtained by the tail bleeding technique: before drug administration, rats were anesthetised with isoflurane and the tip of the tail was cut off; blood samples (80–100 µL each) were then taken from the tail tip at different time points after drug administration. Blood was collected in heparinised tubes, kept in ice and treated as described in [90] within 10 min.

### 3.5 HPLC-UV Analysis

Samples (2 µL) were analyzed by HPLC/UV (1290 Infinity LC System, Agilent Technologies) using a reverse phase column (Zorbax RRHD Eclipse Plus C18, 1.8 µm, 50 x 2.1 mm i.d.; Agilent Technologies) and a UV diode array detector (190-500 nm). Solvents A and B were water containing 0.1% trifluoroacetic acid (TFA) and acetonitrile, respectively. The gradient for B was as follows: 22% for 0.2 min, from 22% to 30% in 1.2 min, then from 30% to 100% in 1.2 min; the flow rate was 0.6 mL/min. The temperature of the column was kept at 35 °C.

The eluate was monitored at 300 or 320 nm, corresponding to absorbance maxima of derivatives (**2** and **3**) and resveratrol, respectively. The absorption coefficients (ε_300_, ε_320_) of resveratrol derivatives, resveratrol and metabolites are very similar. For quantification purposes we assumed the same absorption coefficient.

### 3.6 Statistics

Significance in comparisons was assessed using the Wilcoxon Rank Test.

## 4. Conclusions 

The work presented here shows that sugar groups can be attached to resveratrol, thus making it completely soluble in water - a significant feature - while maintaining to some degree the ability to permeate biomembranes. The next goal may thus be that of producing carbamate derivatives carrying a single substituent on the nitrogen atom, designed to result in a permeant molecule with a lifetime compatible with its intended function as a prodrug.
